# Altruistic preferences of pre-service teachers: The mediating role of empathic concern and the moderating role of self-control

**DOI:** 10.3389/fpsyg.2022.999105

**Published:** 2022-10-28

**Authors:** Maohao Li, Wei Li, Qun Yang, Lihui Huang

**Affiliations:** ^1^Faculty of Education, Sichuan Normal University, Chengdu, China; ^2^Department of Psychology, School of Education, Hangzhou Normal University, Hangzhou, China; ^3^Institute of Psychological Science, Hangzhou Normal University, Hangzhou, China; ^4^Institute of Brain and Psychological Sciences, Sichuan Normal University, Chengdu, China

**Keywords:** pre-service teachers, non-pre-service teachers, altruistic tendency, self-control, empathic concern

## Abstract

Empathy and altruistic behavior are more crucial abilities for pre-service teachers to possess when compared with other study fields. The relationship between empathy and altruistic behavior in Chinese pre-service teachers and their underlying mechanisms, however, has received relatively little attention in the literature. Therefore, the goal of the current study was to examine the links between study fields (i.e., pre-service teachers whose study field is pedagogy and non-pre-service teachers whose study field is non-pedagogy), self-control, emotional empathy (i.e., empathic concern), and altruistic preferences among undergraduates and graduates in five Chinese universities (the age range of participants is 18–20 years; 58.4% women) with the Interpersonal Reactivity Index-C Questionnaire, the Self-Control Scale, and the Chinese Self-Report Altruism Scale tests. The results showed a significant difference between pre-service and non-pre-service teachers in empathic concern and self-control. Furthermore, empathic concern and altruistic behavior tendency of pre-service teachers were significantly higher than those of non-pre-service teachers. Moreover, mediation analyses indicated that empathic concern partially mediated the relationship between study fields and altruistic tendency. Moderated mediation analysis further revealed that self-control buffered the relation between empathic concern and altruistic behavior tendency. These results demonstrate that altruistic tendency of pre-service teachers is influenced by empathic concern and self-control.

## Introduction

InChina, a saying goes, “it takes ten years to grow a tree, whereas a hundred years to cultivate a good man.” Teachers play an essential role in students’ growth and development. Teachers’ altruistic behaviors will affect students, which has an impact on the future development of the country and society. In the current Chinese education system, the primary source of teachers is pre-service students with professional education knowledge in a normal university. In China, normal universities are universities that prioritize teacher education (Wang and Zhao, 2021). Pre-service teachers refer to undergraduate or graduate students whose study field is pedagogy and who undertake a teacher education curriculum to qualify for a degree in education (National Council for Accreditation for Accreditation of Teacher Education [NCATE], 2002). Some studies found that altruistic behavior among youth groups in contemporary society has been weakened (Tettegah and Anderson, 2007; Haddara and Lingard, 2017), which will undoubtedly bring a series of adverse effects on the construction of a harmonious society. Pre-service teachers are a part of the youth group who take the responsibility of educating students, so it is of paramount significance to evaluate the altruistic behavior of pre-service teachers.

Existing studies found that it is more conducive for teachers to become the promoters of students’ learning if teachers can actively perceive students’ emotions and feelings (Viadero, 2004) and even influence students’ later careers and interpersonal communication (Myrick, 2003). Therefore, society has more positive expectations of teachers, requiring teachers to have more altruistic behaviors.

Altruistic behavior refers to a kind of behavior in which an individual would rather sacrifice his interests to meet the needs of others (Fehr and Fischbacher, 2003; Fehr and Rockenbach, 2004). According to previous research, motivations for altruistic behavior include negative emotions (Blair and Mitchell, 2009; Kimonis et al., 2019; Thielmann et al., 2020), a desire for fairness (Fehr et al., 2008; Engelmann and Tomasello, 2019), and moral emotions (de Oliveira-Souza et al., 2016). Altruistic behavior, however, is a decision behavior that demands rational analysis by the brain rather than just being a simple, intuitive behavior. According to neuroscientific studies, the anterior cingulate cortex and the anterior insula are activated when people see other people in pain. The degree of activation in these brain regions significantly correlates with a person’s capacity for empathy. More importantly, the response of the anterior insula and anterior cingulate cortex can accurately predict individuals’ later helping behavior (Hein et al., 2010). In addition, the arousal-cost-reward model proposed by Penner et al. (2005) suggests that when someone’s distress induces the individual’s empathy arousal, empathy arousal will lead to an individual’s negative emotions, which motivates the individual to take some actions to alleviate these negative emotions. Altruistic behavior is one of the ways to achieve this goal. Thus, empathy, an individual’s ability to perceive and understand others’ emotions and respond appropriately (Decety and Svetlova, 2012; Decety et al., 2016), is an essential mediator between individuals and altruistic behaviors (Davis and Kraus, 1997).

Empathy includes both affective empathy and cognitive empathy (Gladstein, 1983). Cognitive empathy is a top-down ability that allows people to think about problems from the perspective of others, primarily including perspective taking, whereas affective empathy is a bottom-up ability that will enable people to perceive the emotions of others, principally including empathic concern (Decety and Meyer, 2008; Heyes, 2018).

Previous studies examined the prediction of affective empathy on altruism (Batson and Coke, 1981; Batson and Toi, 1982; Batson, 1997; Cialdini et al., 1997; Klimecki et al., 2016). Batson et al. (2007) proposed that empathic concern will arise when (a) another person’s welfare is valued terminally, not as an instrumental means to self-benefit, and (b) that person is perceived to be in need. As teachers, when we desire to help students actively, the awakening of empathic concern will allow us to perceive students’ emotions and feelings to better help students. Cialdini et al. (1997) also suggest that empathic concern for others results in selflessness and true altruism. Notably, empathic concern affects helping primarily as an emotional signal of oneness. Namely, teachers are also “students” who have experienced the learning difficulties their students are experiencing. Consequently, they can perceive more of themselves in the other (i.e., their students). In addition, studies on altruistic punishment found that, when subjects had both the choice of “helping the victim” and “punishing the perpetrator,” there was a significant positive correlation between empathic concern and helping the victim (Leliveld et al., 2012; Hu et al., 2015). Studies on teachers’ empathy found that teachers’ ability to empathize is beneficial for addressing school bullying (Tettegah and Anderson, 2007) and is positively correlated with students’ prosocial behavior in self-report (Raskauskas et al., 2010). Moreover, as students grow through life, they will not always take the initiative to tell teachers their current emotional feelings. Therefore, teachers must be able to perceive students’ emotions and feelings accurately. Affective empathy plays a significant role in this process. Consequently, affective empathy is regarded as a necessary trait and competency for normal students relative to students in other study fields, contributing to students’ overall development and teachers’ professional growth (Peck et al., 2014).

Studies on self-control suggest that self-control also plays a vital role in altruistic behavior. Self-control is the ability of individuals to control their consciousness (including thoughts) and behaviors in a goal-oriented fashion (Bowers et al., 2011). As has been argued, negative emotions are part of the motivation for altruistic behavior (Cialdini et al., 1981; Cialdini and Trost, 1998; Jordan et al., 2016b; Kimonis et al., 2019; Thielmann et al., 2020). When the individual as an observer finds that others are in distress, empathic arousal will induce the individual’s negative emotions and lead to altruistic behavior (Cialdini et al., 1987; Nelissen and Zeelenberg, 2009). However, altruistic behavior requires individuals to sacrifice their interests (Jordan et al., 2016a). In this case, it is necessary to restrain selfishness through self-control to act in altruistic behaviors (Müller-Leinß et al., 2018). In addition, there is a need when it comes to self-control to modulate the relationship between negative emotions and altruistic behavior in this process of restraining selfishness through self-control to act in altruistic behaviors (Eisenberg et al., 1998; Grecucci et al., 2013). Therefore, self-control is another factor that mediates the relationship between negative emotions generated by empathy and altruistic behavior (Krueger and Hoffman, 2016; Bellucci et al., 2017). Other studies found moral emotions are also the motivation for altruistic behavior (de Oliveira-Souza et al., 2016). Moral emotions will surface when a person, acting as an observer, notices that others are being treated unfairly. At this moment, individuals need to adjust their moral emotions through self-control to decide whether to make altruistic behaviors or not. In lesion studies employing economic games as surrogates of moral emotions like guilt and envy, participants’ decisions to punish or donate reflected moralistic punishment and generous response inclinations. Unquestionably, their decisions to punish or donate are regulated by self-control (Krajbich et al., 2009). That is to say, the moral emotions aroused by the violations of others will predict the following punishment behavior. If the individual does not want to be retaliated against by others or does not want to lose profits, in that case, it is paramount to control themself to not punish the violator. In addition, from the perspective of the viewer (or witness), the dorsomedial prefrontal cortex integrates representations of intent with the agent’s actual behavior (completed harm vs. no harm at all) to come up with a final condemning or exculpating judgment (Young and Saxe, 2008). It is universally acknowledged that the dorsolateral prefrontal cortex is a crucial brain area for self-control (Miller and Cohen, 2001; Delgado et al., 2005; Knoch et al., 2006), which indicates that self-control modulates an individual’s moral emotions to determine whether to act altruistically. As empathy is one of the moral emotions, self-control should also play a moderating role between empathic concern and altruistic behavior in theory.

**FIGURE 1:**
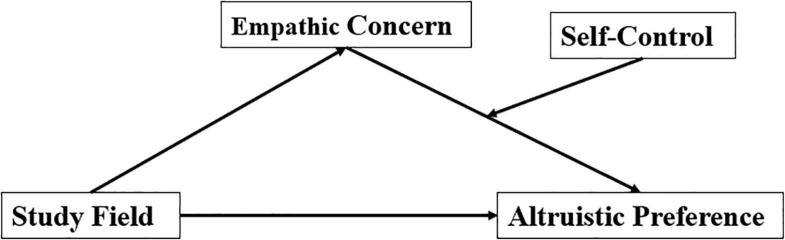
The proposed theoretical model.

Based on the literature review, we propose the following hypotheses: First, compared with non-pre-service teachers, pre-service teachers exemplify more altruistic tendencies and empathic concern; second, empathic concern mediates the relationship between study fields and altruistic tendency; and third, self-control mediates the relationship between empathic concern and altruistic tendency (Figure 1).

## Materials and methods

### Participants

A total of 841 undergraduate and graduate students whose study fields include pedagogy, psychology, literature, sociology, engineering, and science from five universities in China participated in this study. The 4-year undergraduate education system provides sufficient time for pre-service teachers to systematically learn professional theories and cultivate their practical technical ability (Hu et al., 2021). Therefore, these undergraduates whose study field is pedagogy and who are students that are willing to become teachers in future were grouped as pre-service teachers. Participation in the present study was entirely voluntary, and no compensation was given to the participants for their participation. To abide by local government policies, the study questionnaire was distributed to potential participants electronically *via* Wen Juan Xing. This platform provides functions equivalent to Amazon Mechanical Turk (Changsha Ranxing Science and Technology, Changsha, China), and no face-to-face contact was made. All participants consented to participation, and data were anonymized. People younger than 18 years or older than 26 years who did not completely fill out the demographic section or whose answers were all the same were exempted from the analyses. To ensure the validity of the data, we also eliminated the scores of each questionnaire according to three standard deviations. Of the remaining 741 participants, 58.4% were women (*M*_age_ = 21.44, *SD* = 2.06, range = 18–26). Descriptive statistics of specific demographic variables are given in Table 1.

**TABLE 1:** Descriptive statistics of specific demographic variables.

Variable	Options	Frequency	Percentage
Gender	Men	308	41.6%
	Women	433	58.4%
Grade	Freshman	134	18.1%
	Sophomore	133	17.9%
	Junior	120	16.2%
	Senior	91	12.3%
	1 Master	181	24.4%
	2 Master	50	6.7%
	3 Master	32	4.3%
Study fields	Pedagogy	309	41.7%
	Non-pedagogy	432	58.3%

### Questionnaire

#### Interpersonal reactivity index-C questionnaire

The Interpersonal Reactivity Index-C (IRI-C) Questionnaire, which was revised by Zhang et al. (2010) based on IRI (Davis, 1980), was used to measure the empathic ability of Chinese participants. The revised questionnaire consisted of 22 questions divided into four subscales: perspective taking (PT, the tendency to adopt the point of view of other people), empathic concern (EC, the tendency to experience feelings of warmth, compassion, and concern for other people), personal distress (PD, one’s own feelings of personal unease and discomfort in reaction to the emotions of others), and fantasy (FS, an exciting and unusual experience or situation you imagine happening, but which will probably never happen). Individuals rated each item on a five-point Likert scale, with 0 indicating “very inappropriate” and 4 indicating “very appropriate,” with higher scores indicating higher empathy. Cronbach’s alpha of IRI-C was 0.80.

#### Self-control scale

The present study adopted SCS to measure the self-control ability of Chinese participants, which is based on Tangney et al. (2004) Self-Control Scale. Nineteen items were preserved in view of cultural differences and reliability (Tan and Guo, 2008). The scale still was divided into five subscales: controlling impulses (six items, such as “I am too prone to lose my temper”), keeping healthy habits (three items, such as “I am lazy”), resisting temptation (four items, such as “I can resist the temptation”), focusing on work (three items, such as “I can’t concentrate”), and controlling entertainment (three items, such as “I do something that will give me pleasure but do harm to myself”). All items were measured on a five-point Likert scale, with 1 indicating “strongly disagree” and 5 indicating “strongly agree,” with higher scores indicating greater self-control. Cronbach’s alpha of SCS was 0.87.

#### Chinese self-report altruism scale

The Chinese Self-Report Altruism Scale (Li, 2008), which researchers in mainland China frequently use, was adopted in the current study to accurately describe the altruistic behavior of young people in mainland China. There were 22 questions in the Chinese Self-Report Altruism Scale, broken up into five subscales: Responsible Altruistic Behavior, Respect and Care for Others, Care and Focus on Yourself, Altruistic Behavior Performance and Fantasy, and Egoistic Behaviors and Perceptions. Higher scores indicated greater altruism. Participants rated each item on a seven-point Likert scale, with 1 denoting “very inappropriate” and 7 denoting “very appropriate.” Cronbach’s alpha of the Chinese Self-Report Altruism Scale was 0.87.

#### Common method biases test

The current study’s data were gathered using Interpersonal Response Index Inventory (IRI-C), the Self-Control Scale, and the Chinese Self-Report Altruism Scale (Li, 2008). As a result, there might be widespread method biases at play here. To achieve this, the questionnaire design and the response procedure must be strictly controlled, and the data must also undergo a single-factor test for statistical analysis. Specifically, the results of the unrotated principal component analysis were examined by conducting exploratory factor analysis on all items, and a serious common method bias was determined if only one factor or a common factor had particularly high explanatory power (Eby and Dobbins, 1997; Livingstone et al., 1997; Podsakoff et al., 2003); if multiple factors with eigenvalues greater than 1 are obtained and the amount of variation explained by the first factor does not exceed 40%, then the common method bias is not severe (Ashford and Tsui, 1991). According to the test results, there are 15 factors with eigenvalues greater than 1 in unrotated principal component analysis, and the first factor accounts for 18.48% of the variance. Therefore, the current study’s common method bias issue is not major.

#### Data analysis

The objectives of this research were to see whether empathic concern played a mediating role between study field and altruistic tendency in undergraduate and graduate students and, if so, whether self-control played a moderating role in the latter path between empathic concern and altruistic tendency. These research questions were tested in three steps. First, the descriptive statistics and bivariate Pearson’s correlations were calculated. Second, the mediating effect of empathic concern was examined by using PROCESS macro for SPSS (Model 4) (Hayes, 2017). Third, the analyses of the moderating effect of self-control on the latter links between empathy and altruistic tendency were constructed by applying the PROCESS macro (Model 14). All study continuous variables were standardized, and the models utilized 5,000 resamples through bootstrapping confidence intervals (CIs) to determine whether the effects in PROCESS Model 4 and Model 14 were significant (Hayes, 2017).

## Results

### Correlation between study field, altruistic tendency, empathic concern, and self-control

The Pearson correlations are presented in Table 2. The study field was positively correlated with altruistic tendency and empathic concern. Altruistic tendency was positively associated with empathic concern and self-control. Empathic concern was also positively correlated with altruistic tendency.

**TABLE 2:** Pearson’s correlation coefficient.

Variables	1	2	3	4
1. Study field	—			
2. Altruistic tendency	0.14**	—		
3. Empathic concern	0.12**	0.62**	—	
4. Self-control	0.03	0.32**	0.17**	—

Study field was dummy coded as 0 = non-pedagogy; 1 = pedagogy.

**p < 0.01.

### Analysis of empathic concern as a mediator

To test the mediating effect of empathic concern, we used Model 4 of the SPSS macro PROCESS complied by Hayes (2017). The regression results for testing mediation are reported in Table 3. After controlling for gender and age, the results indicated that study field was positively related to empathic concern (*b* = 0.16, *p* < 0.05); in addition, study field and empathic concern were positively associated with altruistic tendency (*b* = 0.14, *p* < 0.05; *b* = 0.61, *p* < 0.001). As the direct predictive effect of the study field on altruistic behavioral tendency was significant (*b* = 0.24, *p* < 0.01), empathic concern partially mediated the association between study field and altruistic tendency. The bias-corrected percentile bootstrap analyses further showed that the mediation effect accounted for 42% of the total effect of the study field on altruistic tendency; the mediating effect was 0.10, with a 95% CI of [0.0148, 0.1894].

**TABLE 3:** Analysis of empathic concern as a mediator.

Regression equation		Fit index			Regression coefficient		
Dependent variable	Independent variable	*R*	*R* ^2^	*F*	*B*	*SE*	*t*
Empathic concern	Study field	0.24	0.06	15.35	0.16	2.19	7.43*
Altruistic tendency	Study field	0.62	0.39	116.83	0.14	0.06	−2.28*
	Empathic concern				0.61	0.03	20.41***

*p < 0.05 and ***p < 0.001.

### Analysis of self-control as a moderator

**FIGURE 2:**
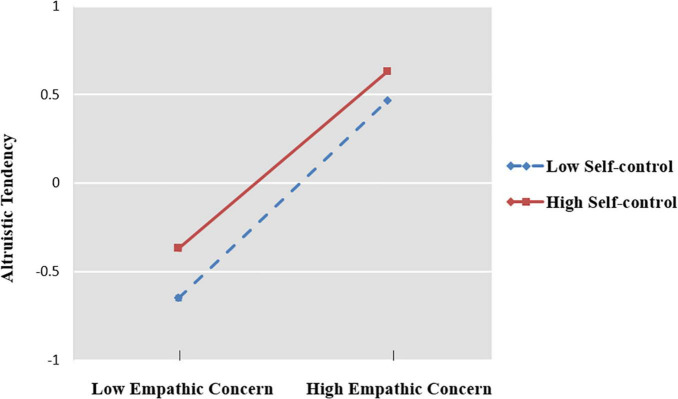
Interaction graphs.

To examine whether the latter indirect relationships between study field and altruistic tendency *via* empathic concern would be moderated by self-control, we used Model 14 of PROCESS macro developed by Hayes (2017). The regression results for testing the moderator are reported in Table 4. The results showed that self-control positively interacted with empathic concern in predicting altruistic tendency. The interaction effect is visually plotted in Figure 2. Simple slope tests revealed that empathic concern had a significant positive effect on altruistic tendency in high- and low-level self-control. The effect of empathic concern on altruistic tendency was weaker for college students with high levels of self-control (*b _*simple*_* = 0.50, *t* = 12.53, *p* < 0.001) than for those with low levels of self-control (*b _*simple*_* = 0.62, *t* = 18.34, *p* < 0.001), and the mediating effects of self-control at different levels are reported in Table 5.

**TABLE 4:** Analysis of self-control as a moderator.

Regression equation		Fit index			Regression coefficient	
Dependent variable	Independent variable	*R*	*R* ^2^	*F(df)*	*B*	*t*
Altruistic tendency		0.66	0.44	96.07***		
	Empathic concern				0.56	19.02***
	Self-control				0.22	7.82***
	EC × SC				–0.06	−2.61**

EC is empathic concern and SC is self-control.

**p < 0.01 and ***p < 0.001.

**TABLE 5:** Mediating effects of self-control at different levels.

	Self-control	Effect	Boot SE	Boot LLCI	Boot ULCI
Level of self-control	47.79 (*M*-1*SD*)	0.62	0.034	0.55	0.68
	58.03 (*M*)	0.56	0.030	0.50	0.61
	68.27 (*M* + 1*SD*)	0.50	0.040	0.42	0.58

## Discussion

The current study aimed to investigate the relationship between study field and altruistic tendency. In addition, it is of paramount importance to check whether empathic concern mediates the relationship between study fields and altruistic tendency and whether self-control moderates the relationship between empathic concern and altruistic tendency. The results revealed that study fields were negatively correlated with empathic concern and altruistic tendency, while empathic concern was positively correlated with altruistic tendency. Pre-service teachers show more altruistic tendencies and empathic concern than non-pre-service teachers. More importantly, empathic concern mediates between study fields and altruistic tendencies. Moreover, we found that self-control moderates empathic concern and altruistic tendency.

In terms of study field differences in altruistic tendency, pre-service teachers have a higher altruistic tendency than non-pre-service teachers, which is consistent with previous studies (Bostic, 2014). First, some studies found that teachers have a high sense of professional identity (Zhang et al., 2016; Wang et al., 2017; Weinberg et al., 2021). According to social identity theory, professionals will identify with both their occupation and their organization at the same time (Dutton et al., 1994; Tajfel and Turner, 2004), and further studies found that employees’ professional identity can promote the generation of their organizational identity (Meixner and Bline, 1989). The term “organizational identity” refers to an individual’s perceptual cognition and emotional sense of belonging to the organization to which they belong. This psychological foundation underscores their preference for altruistic behaviors, such as upholding the organization and assisting its members (Ashforth et al., 2008). Gaziel (1995) found that teachers’ professional identity was significantly negatively associated with their willingness to leave the workplace and their jobs. So teachers’ professional identity will affect their organizational identity. In addition, individuals’ organizational identity is positively related to good interpersonal relationships between individuals and their colleagues (Morgan, 1986). Because of the good internal relationship among pre-service teachers, they are more willing to regard their classmates or colleagues as in-group individuals. According to the in-group favoritism theory (Tajfel et al., 1971; Vermue et al., 2019), people are more likely to assist those who share their identity. Hence, pre-service teachers have a more significant concern for empathy than non-teachers. Second, social desirability refers to the internal psychological tendency of individuals to try their best to make their behaviors and ideas meet the needs of society and the masses, expect to be recognized by society, and maintain their self-image (Perinelli and Gremigni, 2016). From ancient times, Chinese society has expected teachers to be proactive and to go out of their way to help students. According to the social desirability theory, excellent interpersonal desirability will promote individuals’ careers to shape the corresponding self-belief and self-requirement (Carlo et al., 1991; Braun et al., 2001; Lalwani et al., 2006; Wanat et al., 2020; Lanz et al., 2022). Consequently, the expectation of Chinese society that teachers should actively assist students will motivate teachers to meet and maintain this social expectation throughout their academic and professional careers (Xuan, 2006; Li, 2017).

In terms of study field differences in empathic concern, pre-service teachers have higher empathic concern than non-pre-service teachers. Certain emotions always accompany students’ learning. Teachers with high empathic concern can timely pay attention to students, empathize with students’ emotional changes, and adjust their teaching methods simultaneously (McAllister and Irvine, 2002). Coffman (1981) found that teachers with a high empathic concern could communicate with their students in a way that made them feel understood and that their emotions were felt. Students were inspired to change their attitudes toward learning, encouraging them to take the initiative to learn and facilitating their academic success. When teachers pay good emotional and empathic attention to their students during the teaching process, it enables students to develop in the long run. This is another way that teachers’ empathy can enable the students to socialize (Peart and Campbell, 1999). Moreover, to meet pre-service teacher’s own developmental needs, empathic skills are also trained in their daily courses that include experiential training (Kolb et al., 2001), skills training (Redman, 1977), video training (Barone et al., 2005; Liu, 2012; Shin, 2017; Innamorati et al., 2019; Liu and Ren, 2019), and practice mindfulness (Kabat-Zinn, 1990). Pre-service teachers are trained in these daily courses during their academic career, so their empathic attention skills are high compared with other study fields.

Regarding the mediating role of empathic concern, the results showed that empathic concern mediated the relation between study field and altruistic tendency. Previous studies have looked into the effect that affective empathy has on altruism by making predictions about it (Batson and Coke, 1981; Batson and Toi, 1982; Batson, 1997; Cialdini et al., 1997; Lahvis, 2017; Crockett and Lockwood, 2018; Canevello et al., 2021; Miyazono and Inarimori, 2021). In the group of pre-service teachers, empathic concern is a desirable trait for their professional development. They can be trained in empathy through courses and by watching empathy videos (Liu, 2012; Shin, 2017; Innamorati et al., 2019; Liu and Ren, 2019). Eventually, they are easily motivated to perceive others’ emotions and feelings, which makes them more willing to help others and thus demonstrates a stronger altruistic tendency than students of other study fields. Cialdini et al. (1997) ever suggested empathic concern for another result in selflessness and true altruism. Batson et al. (2007) also proposed that empathic concern arises when (a) another person’s welfare is valued terminally, not as an instrumental means to self-benefit and (b) that person is perceived to be in need. Accordingly, the emergence of empathic concern will enable teachers to perceive students’ emotions and feelings to assist students more effectively when they want to help them actively. Compared with pre-service teachers, non-pre-service teachers are not professionally trained in empathy, and empathy is not a necessary trait and ability for them. Consequently, their empathic attention ability is relatively low, and it is not easy for them to empathize with others’ unfortunate circumstances, so their altruistic tendency is low. Previous studies showed that empathy is a crucial mediating variable between individual’s negative emotions and altruistic behavior (Davis and Kraus, 1997). Individuals with high empathic concern are more likely to feel the negative emotions of others and thus are more inclined to help others (Cialdini et al., 1981; Cialdini et al., 1987; Cialdini and Trost, 1998; de Waal, 2008; Hu et al., 2015; Decety et al., 2016). Therefore, pre-service teachers not only have higher altruistic tendencies but also understand and enter into others’ feelings through empathic concern, thereby enhancing their altruistic tendency.

Regarding the moderating role of self-control for empathic concern, the results demonstrate that, for individuals with high empathic concern, the lower the self-control, the greater the altruistic tendency, whereas for individuals with low empathic concern, the higher the self-control, the greater the altruistic tendency. According to previous studies, the factors influencing altruistic behavior include negative emotions (Cialdini et al., 1981; Cialdini et al., 1987; Cialdini and Trost, 1998; Darley and Pittman, 2003; Blair and Mitchell, 2009; Kimonis et al., 2019; Thielmann et al., 2020) and selfishness (McAuliffe et al., 2015; Yang et al., 2018). In the process of altruistic decision making, individuals face a cognitive conflict. That is, the negative emotions that individuals generate when they observe others in unfavorable situations will drive them to make altruistic behaviors, but altruistic behavior requires sacrificing their interests simultaneously. Therefore, individuals need self-control to resolve the conflict between self-interested motives and emotional impulses (McAuliffe et al., 2015; Yang et al., 2018). According to previous studies, negative emotions and motivations to help others aroused by empathy will surpass selfish motivation, so individuals with higher empathy are more inclined to help others (de Waal, 2008; Hu et al., 2015). Therefore, for individuals with a high empathic concern, the lower their self-control ability, the less they are capable of repressing the negative emotions they experience when witnessing the suffering of others and the greater their willingness to engage in altruistic behavior. However, individuals with strong self-control can control their negative emotions and consider their interests rationally when deciding whether or not to engage in altruistic behavior. In contrast, for individuals with low empathic concern, others’ misfortune does not evoke strong negative emotions, and whether or not to help others at that time is primarily influenced by selfish motivations; that is, individuals with low self-control ability are unable to control their selfish motivations, so they are hesitant to engage in altruistic behavior. Individuals with high self-control can control their selfishness, make rational cognitive decisions, and engage in more altruistic behavior. Other studies suggested that moral emotions are also predictors of individual altruistic behaviors (de Oliveira-Souza et al., 2016). Moral emotions motivate individuals to behave altruistically when they notice others being treated unfairly. Nevertheless, whether they will help the victim next is modulated by self-control (Young and Saxe, 2008; Krajbich et al., 2009). If the individual does not want to be retaliated against by the offender or does not want to lose his interest, then he needs to control himself and not punish the offender.

It is essential to be aware of some restrictions on this study. First, in conjunction with previous research, perspective taking, personal distress, and fantasy are also predictors of altruistic behavior (Oswald, 1996; Tusche et al., 2016; Pan et al., 2022). However, in the present study, we did not find a significant role for perspective taking, personal distress, and fantasy in the altruistic behavior of the pre-service teachers. Therefore, it is well worth further exploring the role of perspective taking, personal distress, and fantasy in different groups of study fields in future. Second, in the training courses for normal students in a normal Chinese university, pre-service teachers will be taught how to accurately perceive students’ emotions and feelings to take appropriate measures to help students overcome difficulties. Notwithstanding, this can easily lead to a question: Does learning a particular study field affect empathic concern? The present study did not provide a satisfying response to this question, but future research might examine changes in teachers’ empathic concerns. Third, the cross-sectional design of this study failed to confirm causal relationships between study fields, altruistic tendency, and empathic concern. Therefore, future longitudinal research is required to establish the causal relationship. Fourth, the current study collected data through questionnaires, which may have reduced the results’ reliability. Accordingly, future research could recruit students from different study fields to explore the relationship between study fields, empathic attention, and altruistic behavior through altruistic punishment games. Finally, the sample was limited to college students from a university in the Midwest. Therefore, it is conceivable that the sample does not adequately represent the majority of pre-service teachers in China.

Meanwhile, it should be noted that the current study allows us to discover the relationship and mechanism between empathic concern and altruistic preference in this particular sample. Hopefully, it can also provide some guidance for the future curriculum setting of teacher education.

## Data availability statement

The raw data supporting the conclusions of this article will be made available by the authors, without undue reservation.

## Ethics statement

This study was carried out in accordance with the recommendations of the Ethics Committee of the Institute of Brain and Psychological Sciences, Sichuan Normal University. The protocol was reviewed and approved by the Ethics Committee of the Institute of Brain and Psychological Sciences, Sichuan Normal University (approval no. SCNU-210602). Written informed consent was obtained from all participants for their participation in this study.

## Author contributions

ML was responsible for the current study’s design, data collection, interpretation of data for the work, manuscript writing, and revising. WL contributed to the data collection and data analysis. QY and LH contributed to the revision of the manuscript. All authors contributed to the article and approved the submitted version.
